# Abstracts and Gleanings

**Published:** 1878-03-20

**Authors:** 


### *Abstracts and Gleanings.*

### CASES OF VARICOSE VEINS.

CASE 1. F—— M——, a German, aged 36 years, of large frame, weighing about 220 pounds, called at my office February 13, 1868, to consult me in regard to an amputation of his left leg on account of a varicosed condition of the superficial veins of that leg.

On examination of the limb I found the superficial veins below the knee very much dilated and tortuous, the tension and weight of which caused great pain and suffering. The external saphena was dilated to almost an inch in diameter, while the superficial veins of the thigh also presented a dilated, tortuous, and sacculated appearance.

At about the union of the middle and lower thirds of the tibia and fibula I found a large, foul ulcer, with numerous smaller ones around the joint below. These ulcers had been open for four or five years; the œdema of the limb was well marked, and the course of the veins was much inflamed. During this time many remedies had been used, but the ulcers had persistently refused to heal.

My patient informed me that about twenty days before he came to me, the large ulcer had commenced bleeding, and the hæmorrhage had been so severe that it was with great difficulty it was stopped by the application of cold and compresses. The veins of the opposite limb were very much diseased, with some small ulcers around the ankle joint, but as yet had not troubled him as severely as those in the other limb, although the leg was in a very bad condition.

I did not deem it necessary to amputate, and therefore advised him to wait and give me time to investigate the matter and determine, if possible, the best mode of procedure in his particular case. In referring to Gross', Erichson's and Druitt's works on surgery I found but little difference of opinion expressed by those high authorities in regard to the statement of such cases; each recommending the removal of a part of the vein, pin ligature, cautery, etc., for permanent relief. Knowing that the object of any operation was the obliteration of the vein, and that any procedure was attended with more or less danger on account of the formation of clots, I at once resolved to try the hypodermic injection of sol. of persulphate of iron.

Accordingly, on the 14th, after applying a tourniquet above the point where I wished to operate, and having my esteemed friend, Dr. Woolley, make firm pressure below, I proceeded to inject about 30 drops of the remedy above named into the external saphena about its middle. With the pressure continued, in about five minutes I found to my great satisfaction that a firm, hard clot had formed, which was immovable and unyielding. I now made the same preparation and proceeded to operate upon the right leg. This, however, was not so satisfactory. My syringe had become so corroded with the medicine that it was with great difficulty that I forced a very few drops into the vein. The introduction of the remedy into the vein caused quite extensive inflammation along the course of all the veins, but not sufficient to cause alarm or demand any treatment whatever. The ulcers I dressed with carbolic acid, one part to fifteen parts of glycerine and water.

With this treatment upon my part I left the case in the hands of nature for a cure. On the 5th day of March following, every ulcer on the left leg was entirely healed, the veins reduced, and, as my patient expressed it, the leg was as good as ever.

The right leg, on account of the failure of the first operation, had improved but little, if any; therefore, on the 6th day of March I repeated the operation as before, and in the course of about seventeen days this leg was permanently cured.

CASE 2. G—— M——, American, aged 22 years, also large and powerfully built, a farmer by occupation, called on me June 10th, 1875, to be treated for some "sores," (as he called them) on his right leg, which had been troubling him more or less for five years. On examination I found a case almost precisely like the first, excepting that the diseased condition did not extend above the knee, and there had been no hæmorrhage.

In this case, although the patient was a man of extraordinary nerve and will, yet at times he had been confined to his bed with pain and suffering. This being the sixth case which I had treated by this method, I explained to him the operation, and also the danger attending it, but he expressed himself willing to submit to any operation which would promise any chance for relief, as he was tired of living if he must continue to suffer as he had done for years past.

With this understanding I prepared to operate at once, and proceeded as in the case above reported, assisted by Dr. M. A. Hendryx, of this city (then a partner of mine in practice). I injected about one-half of a drachm of the solution at two different points, and watched the formation of clots, which took place in a very short time. Immediately after the operation he rode to his home, about four miles from the city. I heard nothing more from the case until the third day, when I was summoned in great haste to visit him. I confess I was somewhat alarmed by the hasty summons, knowing, as I did, the danger of the formation of a clot, which, if it passed through the vessels and reached the heart, might cause serious trouble if not instant death. I was much relieved on arriving at the bedside of my patient and finding that he had had a severe chill, which alarmed him and his friends. About four hours after he left my office on the day of the operation, he was attacked with severe pain in the limbs, which began swelling quite rapidly, and on this, the third day, he had a severe chill, followed by a high grade of fever.—DR. F. B. WOOD, in *Detroit Med. Jour.*

---

BACTERIA, AND WHAT WILL KILL THEM.

In the *Circular* of January last was an article upon "Bacteria," by Prof. Tyndal, which presented a remarkable view of that microscopic vegetable in the production of the phenomena of fermentation. Below we present a short communication that appeared recently in the New York *Times*, which shows their wonderful tenacity of life, and what will not and what may destroy their life.

One of the most active and dangerous forms of bacteria, the micrococcus, is about the shape of the head of a small pin; or, rather, when magnified 800 times, it looks like this: o o o o o. Another, the true bacteria, or rod-like particles, are about the following size and shape: 

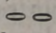

But the principal point is to find out what substances or medicines will destroy them. Quinine will not, for bacteria will live and flourish on a solution of twenty grains to two drachms of fluid. Nor will camphor, for they live on a solution of thirty grains in two teaspoonfuls of fluid. For five days they were seen swimming about among the pieces of camphor, and increasing immensely in numbers. Ten drops of carbolic acid in two drachms of fluid will not kill them. They also flourish in a solution of tar, and will swim about for six or more days between particles of ten grains of calomel in two teaspoonfuls of fluid. One drachm of laudanum in two teaspoonfuls of fluid filled with bacteria will only commence to benumb and kill them at the end of six days.

They lived for ten days in a solution of tincture of nux vomica in two drachms of bacteria fluid. Tannin is the first remedy which has a decidedly destroying effect upon them. It will kill them in two hours; and although they will come to life again after being frozen in ice, and boiled in hot water, yet they will not do this after tannin is applied. Chloroform seems to kill them, but they will come to life again. They will live in a solution of one drachm of chloral in two of water. A concentrated solution of copperas, or sulphate of iron, will kill them; also chlorine water, and dilute muriatic, sulphuric and nitric acids. We may draw the inferences that quinine, calomel and carbolic acid are useless in diphtheria. That opium, nux vomica, chloroform and chloral are comparatively so; and that tannin, sulphate of iron, chlorate of potash, chlorine water, and dilute mineral acids may prove the only really useful remedies.—*The Druggists' Circular and Chemical Gazette.*

---

ACUTE CATARRH OF THE MIDDLE EAR, CAUSED BY SNUFFING WATER UP THE NOSTRILS.

No further proof is needed of the danger of introducing fluids into the nostrils, and the following case is reported only because its features are so clearly marked. The fact that the patient had already lost the function of one ear in a similar way gives additional value to his testimony. The use of the nasal douche and its modifications has been much debated, and is still a question of controversy. It is freely granted that it may be used by the surgeon himself upon hundreds of cases without harm, as has been shown in a recent paper by Cassells. The only point ever sought to be established by the writer is that this therapeutic measure cannot be safely entrusted to patients. The very best of them are often incompetent to remember and carry out the various "precautions" given them, and, in this sense, it is impossible always to tell before-hand that your case is a "proper" one to be allowed to apply the remedy for himself. Moreover, in some cases, harm has certainly resulted where every direction was followed by the patient, and even where the operator was an educated physician. Thus a certian small percentage are constantly suffering injury, which to one placing a high value upon an ear, is not compensated for by all the advantages which this treatment of naso-pharyngeal catarrh is supposed to offer.

Mr. F. was snuffing water up his nose on account of nasal catarrh, and, while blowing it out again, he "felt a drop enter the right ear." Had pain in that ear immediately (this was at noon,) which increased in severity, and at midnight was unbearable. Took large doses of morphine with only partial relief. Next morning he applied for advice, and was found to be suffering great pain, with some fever. The right auditory canal was red and swollen at the bottom, and the drumhead very red and somewhat bulged outwards. H. D. was $\frac{0}{40}$. A paracentesis of the drumhead was made at once, and the hot douche ordered to be used every hour. Immediately after the operation the pain disappeared, and did not return until five days later, when it was relieved by leeching. Under the usual treatment the inflammation subsided, and the hearing was restored. The water which was used in the nostrils was cool and not medicated.

Four years ago this patient felt water enter his left ear while using the nasal-douche, and had acute catarrh of that ear, followed by suppuration and deafness. The H. D. on that side was $\frac{0}{40}$, and the drumhead was cicatricial.

---

RAISING THE ARM IN EPISTAXIS.

Having tried this plan on one or two occasions effectually, I naturally sought for an explanation of the success of a method apparently so empirical. The reason of it at length appeared to me both simple and interesting. It was this: In holding the arms up above the head—for in many cases I did both—the scapulæ are elevated and rotated outwards, and by this means, extension is made upon the ribs by the serrati magni muscles; the chest, thus expanded, causes an increased flow of blood from the venous or right side of the heart to the lungs, and *pro tanto* from the head, and a temporary or partial stasis or diminished flow of blood to the left or arterial side of the heart, thus reducing the *vis a tergo*, and allowing to such extent, therefore, time for the blood to coagulate in the vessels of the nose.

This explanation was confirmed by an observation which just reverses the condition of things. A patient came to me suffering from occasional attacks of hæmoptysis, and spontaneously remarked "that it was sometimes brought about by raising his arms above his head, as in removing anything from a shelf or otherwise." This statement beautifully fitted in with my views, and struck me at once as a remarkable confirmation of what before might be taken only for a possible or plausible explanation.—R. W. ELLIS, in *The Lancet, Nov.* 17, 1877.

### CHLOROFORM HALLUCINATION.

Dr. Folsom, in the *Medical and Surgical Reporter*, says: I saw in the *Medical and Surgical Reporter*, of December 29th, 1877, the report of a case of chloroform hallucination. The following is my experience in the same line: In 1854, a clergyman's sister came to my office for the purpose of taking ether and having a tooth extracted, and brought her brother's wife with her. I began to administer the ether to the patient, and whilst renewing it she got away from me, and seemed alarmed and offended. I did not attempt to compel her to breathe any more ether, but urged her to take it, and so also did her brother's wife, but she would not take any more. She had the impression, so her brother told me, that I attempted to violate her, and that his wife assisted me. It was a long time afterward before she would fully give up that she was mistaken in the matter.

Portsmouth, N. H., Jan. 1, 1878.

### ACTION OF PILOCARPIN ON THE EYE.

M. Galezowski, in a communication to the *Societe de Biologie*, narrated the results of the trials he had made on the eye with pilocarpin, the active principle of jaborandi. These show it to be possessed of powerful myotic powers. One drop of a mixture, consisting of ten parts of water and one-fifth of a part of pilocarpin, instilled into an eye, the subject of paralytic mydriasis, gives rise to such a contraction of the pupil that at the end of half an hour, this measures scarcely a millimetre in diameter, the contraction continuing for from five to eight hours. This result has been verified upon a great number of patients, so that it may be now stated that pilocarpin possesses myotic powers as active as those of eserine, while it does not excite irritation like that substance, the prolonged employment of which may give rise to peri-orbital pains, intense conjunctivitis, and great nausea. M. Galippe observed that the experiments which he and M. Bochefontaine had ma e were attended by precisely the same results as those described by M. Galezowski.—*Gaz. des Hop.—Med. Times and Gazette.*

### THE PANCREAS IN DIABETFS.

M. Lancereau laid before the Academie de Medicine some specimens exhibiting extensive lesions of the pancreas in subjects of diabetes, and having related the histories of the cases whence they were derived, and referring to cases already on record, went on to say that it was thus evident that, at least in some cases, diabetes is accompanied by great alterations in this organ. In these cases the progress of the disease has been relatively rapid, and has been attended by polyphagia, polydipsia, excessive emaciation, and abundant glycosuria—in fact, by all the characteristics of saccharine diabetes. So, also, animals from which the pancreas has been removed, became voracious and rapidly emaciated, and die very quickly. There would seem, therefore, to be no doubt that there is a casual relation between these changes in the pancreas and the disease in question. This form of disease may be distinguished by the relatively rapid occurrence of emaciation with polyphagy and polydipsia and by the peculiar character of the alvine evacuations. Its prognosis is most unfavorable; the indication for the treatment consists in suppressing alimentary substances that are digested by the pancreatic juice, in favor of those which undergo digestion in the stomach.—*Gaz. des Hop.*

### HORSE FLESH.

During the first six months of 1877, the Butchers, who deal in this commodity in Paris, have delivered to consumption 5,283 horses, donkeys, and mules, which furnished 959,730 kilos of meat (net). During the corresponding period of 1876, the number of these animals was 4,422, which gave 803,500 kilos. The increase is, therefore, of a marked character. The persons who endeavored to popularize the use of horse flesh affirm that it is more wholesome and more nourishing than beef, although often not quite so agreeable. Paris contains more than fifty butchers' shops specially devoted to this article. A very good pot-au-feu may be made with the inferior morsels at the price of twenty-five centimes and thirty centimes per pound. The choicest pieces—fillet, undercut, etc.—are much higher in their cost.—*London Veterinary Journal.*

### INFLUENCE OF IRON MIXED WITH FOOD ON THE BLOOD.

Nasse fed a dog weighing about seventeen and two-thirds pounds, during eighty-seven days, with bread and potatoes, giving at the same time, for twenty-five days, fifteen and a half grains of lactate of iron daily, and for the remaining sixty-two days eighteen and a half grains of oxide of iron each day; the dose in each case being mixed with about six-sevenths of an ounce of fat. The weight of the animal increased by more than 2 pounds. The specific gravity of the blood rose from 1052 to 1060.8; that of the serum remained nearly unchanged. The amount of iron in the blood increased from 0.477 per mile to 0.755. The increase of the solid constituents depended solely on that of the blood corpuscles. The amount of iron in the blood rose regularly. In conclusion, the author expresses his belief that it is productive of the most fruitful results.—*British Med. Jour.*

### A CURIOUS DISCOVERY IF A REAL ONE.

According to the London *Sanitary Record*, "Dr. Tschamer, of Gratz has discovered that a fungus grows upon the skins of apples and oranges, precisely similar to the fungus which forms the peculiar germs of infection in whooping-cough. He writes that on oranges and apples which have been kept some time may be found dark brown and black specks, which, when scraped off, appears as a damp powder. Under the microscope this powder is seen to consist of the spores of a fungus, identical with those of the whooping-cough. Taking two of these specks from the skin of an orange, Dr. Tschamer introduced them by a strong inhalation into his lungs. The next day tickling of the throat began, which gradually increased, until, at the eighth day, a thoroughly developed whooping-cough set in. Should the discovery be confirmed, there is an additional reason to see that children abstain from eating apples with the skin on, and from chewing orange peel, which many are so fond of doing."—*Boston Journal of Chemistry.*

### TREATMENT OF SCARLATINA.

In the New York Academy of Medicine, Dr. Thompson said:

Probably in the majority of cases of death from scarlet fever, death was due to true septicæmia, rather than to poisoning by the specific agent of the disease. Bromine being far more powerful as an antiseptic than chlorine, was used both locally and internally in the treatment of diphtheria. Although the doctor had not much reason to think that the chlorine treatment was not as successful as possible perhaps in the treatment of scarlet fever, yet he had of late relied upon the use of bromine. Since he had adopted its use he had not seen cases of lymphangitis, and there was a full concurrence in the remarks of Dr. Smith, to the effect that the throat complication, the swelling of the glands about the neck, etc., had been much less frequent since the internal as well as the external use of antiseptics.

The manner of using the bromine was as follows: Dr. J. Lawrence Smith's solution was employed, consisting first of a saturated solution of bromide of potassium in water oz. ij, to that oz. i. of bromine was added very slowly, shaking the water constantly while making the combination. It was better to add half of the bromine first, and then let the bottle stand for half an hour or more before the remainder was added. When the bromine was dissolved in that manner, the bottle was to be filled with water until a *four ounce* mixture was made. The solution thus prepared could be again prepared for administration by combining it with water in any proportion desired.

For internal administration dr. i. of the solution to oz. i. of water was used, and of that a teaspoonful was given in a tablespoonful of sweetened water p. r. n. The solution should be kept in a dark place. As a local application, equal parts of the solution and glycerine could be employed, or in serious cases, the solution could be applied clear. The odor of diphtheria was entirely destroyed by the bromine solution.

With sudden onsets of the disease and a temperature rising rapidly to 104°—105 or 106°—wrap the child in warm blankets and apply douche to the head. Also when the temperature reached 104° the cold, wet pack was recommended with the statement that he had never seen any harm follow its use.

The cold bath was not recommended; the cold wet pack was believed to be all that was necessary. Take a sheet, wring it from water at the ordinary temperature, wrap the child in it, and over that lay one wrung from ice water. The prompt manner in which patients had improved under such management had led him to regard the wet pack as one of the great therapeutic resources for that class of cases.

From the very commencement of the disease the body should be oiled over three times a day. One reason was because it was the most effectual means of relieving the itching of the skin and the excessive restlessness of the patient. Another reason was because it was truly antipyretic—it reduced the temperature. Another reason was because of the close sympathy existing between the skin and kidneys; the oiling kept the glands of the skin in an active condition.—*N. Y. Med. Record.*

AMPUTATION BY LIGATURE.

Dr. Bitot (*Le Progres Medical*, December 15, 1877) advocates the use of the ligature in certain cases of amputation of limbs. The bones, as well as the soft parts, may be thus divided. Disarticulation is accomplished more rapidly than amputation in the continuity of the bones. So long as the epiphyses are not united, separation of the limb at the point of junction with the diaphysis is easily accomplished—a less dangerous operation than disarticulation.

Of course, the ligature is not expected to replace the knife in ordinary cases, but cases do occur in which the patient absolutely refuses the latter. In such cases, the practitioner will be glad to have another means at his command, however defective it may be. Dr. Bitot relates an instructive case, which will be followed by others. The case was that of a man born in 1811:

In 1851, he had a fall from a horse, in which the right knee was seriously contused. This was followed by great pain and stiffness of the joint. In 1854, a small tumor was noted in the articulation, which did not yield to ordinary treatment. The tumor enlarged, and was finally pronounced to be malignant. Amputation of the thigh was advised, and the operation performed in November, 1857. The disease returned in the sump, following a severe injury, eight years afterwards. The patient absolutely refused re-amputation. The growth was of fungous character, and alarming hemorrhages occurred on several occasions. In November, 1866, more abundant hemorrhage occurred. This was arrested by passing a new cord, of the diameter of an ordinary pencil, around the stump about an inch above the growth. This was made tight enough to arrest the circulation below it; ice was applied, which arrested the pain caused by the pressure of the ligature, and, at the same time, kept the latter soft, and facilitated its passage through the tissues. The cord was tightened from time to time; thus it sank deeper into the flesh, and was followed by cicatricial tissue. At the end of fifty-seven days, the diseased mass was removed with the knife and scissors, very slight bleeding occurring. In eight days, the patient was up, and has remained in perfect health ever since. During the course of treatment, and for three years afterwards, the patient took twenty-five or thirty drops of perchloride of iron in sweetened water, in three doses daily.—*St. Louis Clinical Record.*

---

THE ICE WATER TREATMENT OF CROUP.

A physician—Dr. Maunsell, of Yorkshire—urges the treatment of croup according to this method in the *British Medical Journal.* He illustrates it with this case:

I was called upon to visit a boy, eight years of age, suffering from "croup," or, to adopt our nomenclature, "acute laryngo-tracheitis, with 'croupous' respiration." He had been at school the day previous, though slightly ailing, but had gradually become worse. I had come provided with an emetic mixture of sulphate of zinc and ipecacuanha wine. This was administered twice, at intervals of ten minutes, until vomiting was produced. A bladder had been provided, and, being conveniently filled with ice-cold water, was applied to the throat and upper part of the chest, kept in position well under the chin—the child, of course, being in the prone posture. The child, who naturally objected at first to the cold, became quieter, and, in a short time, fell apparently into a doze. The respiration, which had been harsh and crowing, gradually became less so; the cough lost somewhat its singing sound, and the skin, which had been hot and dry, became cool and moist. I remained for two hours in the house, and when I left, I gave instructions that a large, warm poultice was to be applied for an hour in place of the bladder, and then the iced water to be again applied, alternating so each hour until my return. In about ten hours, I came back and found everything progressing favorably. The child is now well, and I believe the result might have been very different; at any rate, the chances of recovery much diminished, had that treatment not been adopted.—*Med. and Surg. Reporter.*

---

HOW TO INTRODUCE THE HYPODERMIC NEEDLE.

Dr. S. J. Allen, of White River Junction, Vt., writes: Placing the palm of the left hand beneath the patient's arm or leg, with the thumb and fingers draw the skin tight over the upper aspects of the limb, in which state you have an unyielding integument. Then holding the instrument with the thumb and index finger of the right hand, place the bevelled side of the point upon the place you have selected at a proper angle with the surface; then, with a quick forward movement of the thumb and index finger only, keeping the hand immovable, thrust the point through the skin into the subcutaneous tissue. This maneuver, if done quickly, will inflict little if any pain, and the patient will thank you, especially if he has been previously subjected to the almost universal method of pinching up a fold of integument for the puncture.—*Med. Record.*

---

THE BLOOD IN DIPHTHERIA.

MM. Bouchutt and Dubrisay communicated to the Paris Academy of Science (*London Med. Record*) the results of the counting of the blood-corpuscles in diphtheria. The numerations were made by Hayem's process; and the writer proved that in diphtheritic angina the number of white corpuscles is considerably augmented, whilst that of the red corpuscles is diminished. The increase of the white corpuscles vary directly with the gravity of the disease.—*Clinic.*

